# The Future of the Hospitals

**Published:** 1910-03-26

**Authors:** 


### SPECIAL ARTICLE.

### THE FUTURE OF THE HOSPITALS.

### CO-OPERATION WITH THE STATE AND LOCAL AUTHORITIES.

At the annual meeting of the Home Hospitals Association, held on Thursday, March 17, at 16 Fitzroy Square, Sir Henry Burdett, in moving the adoption of the report, dealt at length with the future of the hospitals, and with the whole problem of hospital reform, economics, and administration. Medical relief thirty years ago and to-day, he remarked, showed little development, and the increased popularity of the voluntary hospital tended rather to add to than to decrease hospital abuse. If there has been any change it has rather been for the worse, so far at least as increased thrift and self-help among the patients are concerned. It may be useful to emphasise the difference between the function of the Poor Law and those of the voluntary hospital. The function of the Poor Law is the relief of destitution, while it should be the object and duty of every voluntary hospital to apply its resources entirely to the prevention of destitution by stepping in to grant free medical relief to the provident and thrifty when, through no fault of their own, they meet with accident or are overtaken by disease. Unfortunately, owing to want of co-operation, there is much overlapping of the medical work done by the Poor Law and that done by the voluntary hospitals at the present time. The issue of the Poor Law Commission's Reports, and the fact that we have to face some very momentous changes in regard to all the systems of relief, give an opportunity to face the whole question of the provision of hospital relief for all classes in this country and to meet it on proper lines.

### The Minority Proposals.

Everyone who has read the report of the Majority and that of the Minority of the Poor Law Commission must have recognised that the proposals of the Majority, so far as medical relief is concerned, are the least satisfactory portion of the Majority Report. The Minority proposals, on the other hand, are hardly more satisfactory. They involve the handing over of the whole medical relief of the poor, in-patients, home-patients, and out-patients, to the health authorities. As at present constituted the health authorities are the only public authorities who have never recovered from the persons to whom they have rendered service the costs of such service, or, in any case, the recoveries have been so small as to be virtually inappreciable. If the proposals of the Minority Report are given effect to, three things must ensue in course of time:

Firstly. There will be a Government Medical Service which will have a monopoly of the free treatment of the poor.

Secondly. All medical provident clubs and institutions will be undermined and destroyed owing to the fact that side by side with their contributory arrangements there will be free medical relief.

Thirdly. If the ratepayer is to be heavily taxed to provide gratuitous medical relief for the wage-earning classes, subscriptions to the large hospitals must fall off, and these institutions will either have to be closed or turned over to the State.

### The Question of Expense.

Assuming that such changes are to ensue, it may be well to ask what would be their cost? One witness before the Commission put it as high as twenty million pounds a year, and the lowest estimate is five million pounds; but probably fifteen million pounds is a fair estimate. Let us be quite fair to the Minority Report, which is a most able document, most interesting, and displaying evidence of great courage and energy on the part of those who are responsible for it. But the medical relief proposals contained in this report require the closest consideration. They are dangerous, and they would be impracticable. As the *Times* expressed it in a leading article in its issue dated March 16 last, "If you offer people, as the Minority Report does, relief without any drawback, it is absolutely certain that many will take advantage of it, and that more and more will follow. The thing always grows, and one result will be to undermine all the provident organisations resting on self-help and mutual aid." In the case of London that would mean a financial crisis so far as the municipalities are concerned. London, through its voluntary hospitals alone, roughly relieves two million patients a year, expends a million pounds annually upon such relief, and maintains ten thousand beds in its hospitals for in-pateints. If the effects which the *Times* predicts should the Minority proposals be carried out were to follow, it is perfectly certain that the rates in London and throughout the country would be so materially added to that the present habit of living as far as possible outside urban communities would be extended, and there would arise a crisis in municipal finances which those who so glibly and lightly propose these immense changes, the cost of which will have to be defrayed out of the rates, seem to have little regard for. The expenditure of these additional millions each year under the system proposed in the Minority Report would not only not improve the present medical and hospital arrangements, so far as efficiency and the prevention of hospital abuse are concerned, but would still further tend to pauperise the population and increase the confusion and disorganisation which at present prevail in medical relief work.

### Co-operation.

What is wanted is a scheme of co-operation. The proposals of the Minority Report should be reconsidered upon their merits, and the outline scheme at present suggested should be changed to one embracing the only possible safeguard which can be held in dealing with this question—co-operation. "The opportunity," proceeded Sir Henry, "of the moment is one for a statesman, who is also a man of feeling and a business man. It affords an opening for the introduction of a perfected, up-to-date, exhaustive, and complete medical hospital and nursing service, providing for all classes, presenting throughout the vital principles of thrift and self-reliance with the maximum of efficiency and the minimum of cost. Such a service, so organised, which would embrace and bring into working co-operation every existing agency for medical relief, however supported and however administered, must at once render an immense service to humanity by lessening the sufferings which are caused to masses of the population in towns owing to their ignorance and lack of opportunity to learn how to find exactly the relief they require, as and when they require it. The key-note to success is co-operation."

### How to Secure Co-operation.

If a great voluntary hospital desires to prevent abuse and to enforce a system whereby such abuse shall be avoided, though the best interests of the patients shall always be safeguarded, it is met with difficulties whichever way it approaches the question. A patient applies at the hospital, and is admitted. It appears that the circumstances are such that he could well afford to pay a reasonable sum for his maintenance in the hospital. The hospital authorities are at once in this difficulty: that, medically, it may be inhuman to send him out; practically they have no paying ward to which they can transfer him. In the out-patient department you may find a large number of patients whose ailments are not severe, and who could be well, and indeed better, attended to by a private medical attendant, or a provident institution. At the present time there is no system of co-operation, no intercommunication, and no means of transferring the patient from the hospital to the doctor or the agency which ought properly to undertake his relief and care. The one remedy for the ever-increasing numbers of patients who flock to the out-patient departments of our hospitals, largely to their undoing, is that every hospital should agree to abolish the out-patient department altogether. There is no doubt that in cities the means of medical relief of all kinds are sufficiently great to enable everybody to be provided for properly and adequately. To abolish the out-patient department, to put all applicants for out-relief through a splendidly organised and ample casualty department, which every hospital should maintain; to treat every case there once, and then to send it from the hospital to the private practitioner or the provident dispensary, or, in cases of severer nature, to arrange that the patient shall come again to the consultation department, which every great voluntary and other hospital should possess, where these severe cases should not be hurriedly rushed through in hundreds, but where there should be abundance of time for the best brains and skill of the honorary medical staff to deal with each case on its merits, and to do the best for every patient; these relatively simple adjustments—and it should prove a very simple adjustment administratively, although it is a great revolution in our system of medical relief as it exists to-day—would, without injury to anyone, effect great benefit, and become immensely popular as soon as it got thoroughly into working. The time occupied in introducing such a system and in getting rid of the present anomalous and much-abused, inadequate, and worn-out system will not be more than from six to twelve months, and it will result in enormous benefits, not only by increasing the comforts and advantages of treatment which the hospitals will then be able to offer to all patients, but by giving every patient an opportunity to exhibit a measure of thrift and self-dependence which is sadly needed at the present day.

### In-Patient Reforms—The Foreign Example.

As to in-patient reforms, there is one which would also embrace the Poor Law institutions, and might well be adapted with facility. Let us take an example from the Continent and the United States. There the hospitals classify all their patients. They have wards which commence with those in which the patients pay a remunerative rate for the relief and medical treatment they receive. They have two other classes of wards, (1) in which the patients pay an inclusive rate, which covers all the expenses and the medical charges; and (2) where the medical attendance is unpaid for, but the maintenance charges are paid by the patient. They have a fourth class of wards to which all patients that we know as Poor Law cases, being acutely ill, would be sent. To these latter wards the patient is admitted as a free case, so far as the individual is concerned, but here every patient is paid for by the municipality, or by the County Council, or whatever the local authority may be who is responsible for the maintenance of the provision for the sick. There would still be in this country, as there are already, a number of patients sent into the voluntary hospitals that ought properly to be treated as Poor Law cases. Some Guardians under the present Poor Law pay a small subscription, and in case of accidents and other severe illnesses they sometimes pay the voluntary hospital a special rate for treatment. But really there is no co-operation between the Poor Law and voluntary hospital authorities, and in practice there is often considerable overlapping and some friction. Now, if we had a system of co-operation, one great result would be to bring all the medical buildings which are available for the treatment of disease into perfected relations, so that whilst we retained the present system of administration, the Poor Law—then the Public Assistance—on the one hand, and the voluntary hospital system on the other, the whole number of hospital beds would be made available for the use of patients of all classes who needed hospital care. This could readily be done by a Conjoint Council of representatives of all the medical relief authorities, which would have a thorough knowledge of the whole of the hospital provision. This Council would in that way be able to let it be known in every part of a great town like London at what hospitals there were vacant beds on a given day, for example, and how the patient could most readily obtain the treatment of which he had need. At the present time a patient may go from hospital to hospital without knowing where there is a vacant bed, or when there is likely to be one for his use. The present absence of inter-communication is the cause sometimes of avoidable deaths, and certainly of a large amount of avoidable misery and suffering every week in a great city like London. What is required is a system of co-operation which would gather together all the organisations for indoor and outdoor medical relief in all large cities throughout the country. It should include all the nursing agencies, district nurses, and others having the care of the poor; and it should further provide that the patients should have the fullest knowledge of the provision which was made for their accommodation and comfort so that they might be saved as much time and expense as possible, and that every facility might be afforded them to be provident and thrifty if they can afford to be so, and to obtain the medical relief they require promptly and with the utmost efficiency.

### INSURANCE.

"There are certain essentials," proceeded Sir Henry Burdett, "which we should have to secure in regard to such a system of co-operation. One is insurance. We must have an insurance scheme, and as the Government have told us that they are engaged upon some industrial insurance scheme, I hope they will include in it a provision for hospital care in case of illness for those members of the community who may need it on payment of a moderate rate. It has been proposed to me over and over again that we should have a Hospital and Sick Attendance Insurance Company, which would provide that every person might take out a policy on payment of a premium of so much per annum, which would entitle him to so many weeks' hospital care, and so many days' or weeks' attendance as an out-patient. Actuaries have gone into the scheme, the figures have been worked out and carefully considered, and it turns out that for a sum which is the equivalent of the payment under an ordinary accident policy it is possible, on a sound financial basis, to provide by insurance for the medical treatment and residence in a hospital of patients under the best conditions. The scheme has not gone forward because I have never seen my way to overcome an initial difficulty which presents itself, and that is this. At the present time we have practically no pavilions for the reception of paying patients in connection with our hospitals or elsewhere in this country. To provide such ward pavilions would require initially a large capital sum, and that sum has been estimated for insurance purposes at a quarter of a million at the outset. I expect before we could really cover the requirements of the country it would exceed probably four times that sum. Now, leaving this point for a moment, if the Government is coming out with a system of insurance, it can very well arrange for policies at reasonable rates to be issued through the Post Office or through the insurance offices if they are going to have Government insurance offices which will provide the policyholder with the hospital care and hospital treatment which I have referred to. If they do that we are next brought to a further matter of great importance in reference to co-operation, namely, finance; and it will mean that a complete system of co-operation for the purpose of all medical relief throughout the country must secure adequate payment for medical attendance as well as for hospital maintenance. You cannot expect the doctors to go on getting smaller and smaller fees, and attending more and more patients for nothing, so that any new system which we may set up must provide for the adequate payment of the medical profession for the work which they may be called upon to do."

### MAINTAIN THE VOLUNTARY SYSTEM.

We want then complete co-operation and the welding together of all agencies for medical relief, State and voluntary. Such co-operation can be secured by a little patience, for the time is ripe in the sense that everybody realises that the present state of affairs cannot continue unchanged. Now it is very important that we should maintain the voluntary system of hospital support. That voluntary system has produced a system of hospitals which for efficiency, equipment, administration, and on the whole economy too, is unsurpassed by any other hospital system in the world. It represents a great revenue, and the vitality of the system has been never better revealed than during the last ten years, when a sum exceeding ten millions sterling received from voluntary sources has been spent on new hospital buildings, whilst the revenue of the voluntary hospitals has been increased by many hundreds of thousands of pounds per year. But the time has come, having regard to the abuses and evils of the existing want of system, and for other reasons, when possibly there may be a measure of State aid or State grants to the voluntary hospitals for building purposes. That principle has been admitted in this country, and applied to the Royal Infirmary in Manchester, where the Corporation has contributed more than once to the Building Fund of that institution. By following this example local authorities might properly and gladly come forward and contribute the capital sum necessary to provide the pavilions for paying patients in connection with the voluntary hospitals which are essential to the carrying out of a perfected system of medical relief for the whole country. Another change has recently taken place in London which shows that the trend of public opinion and municipal action is all in the direction of making grants for the benefit of the population who frequent our hospitals when it can be shown to be needed to increase efficiency. The Education Authority in London has recently commenced to make grants to some medical schools attached to the voluntary hospitals, in order to provide that London, as the metropolis of the Empire, shall have a system of medical education which will be worthy of that Empire. These medical schools have been sadly in want of funds in recent years, and this action of the municipality is one which everyone will welcome, and which is calculated to be attended with the very best results.

Hospital Abuse.

The curse of free medical relief in this country has been to tend ever to increase the ease with which the people degenerate more and more towards pauperism. The free relief at the hospital, so readily given in such excessive volume, has lessened the feeling of independence, and made people come to regard the big voluntary hospitals as palaces of pain which they have a right to enter without shame to receive free treatment and medicine, quite irrespective of their social condition and capacity to pay a doctor of their own when they are ill. A system of co-operation which brings into direct relation and the closest intercourse for administrative purposes the whole of the agencies for the relief of the sick in London and throughout the country will speedily alter this demoralising and dangerous state of affairs; and if we have voluntary hospitals with classified pavilions into which all classes of patients could be received, by payment or as free cases; if we have out-patient departments which consist simply of a casualty department where every applicant is seen on the first visit; and a consultation department, to which those cases would be referred who are seriously ill and require medical attention which they cannot obtain in the ordinary way from a private practitioner or a provident dispensary; if we have the closest relations between the voluntary hospitals and agencies and a modernised, humanised Poor Law system of medical relief—if we have these arrangements the whole condition and well-being of the people of these islands will be immeasurably improved. In a few years' time people will read with amazement of the want of organisation, the want of method, the carelessness, the overlapping, the ruinous waste of money, and the many other evils which attend our present system of hospital and medical relief, which have crippled its efficiency and made it one which has been the astonishment of the world. We shall never complete any changes which will be adequate unless they are accompanied, in addition to co-operation and reorganisation, with a system of assurance whereby those who wish to be thrifty, though not blessed with large means, may, by a premium within their means, place themselves in a position to enter the paying wards of a reorganised and reformed voluntary hospital, and so to pay the relatively small cost for hospital treatment.

